# Hierarchical mechanical patterns in morphogenesis: from mollusc shells to plants, fungi and animals

**DOI:** 10.1098/rsif.2024.0918

**Published:** 2025-06-25

**Authors:** Derek E. Moulton, Alain Goriely, Régis Chirat

**Affiliations:** ^1^ Mathematical Institute, University of Oxford, Oxford, UK; ^2^ CNRS, ENSL, UJM, LGL-TPE, UMR 5276, Université Lyon 1, 69622 Villeurbanne, France

**Keywords:** dynamic wrinkling, developmental biology, biophysics, mathematical model, molluscs

## Abstract

Hierarchical patterns, made up of subunits of different sizes intercalated with each other, are present in diverse organisms spanning the plant, fungi and animal kingdoms. Despite these structures appearing in different kingdoms, at different scales, at different levels of biological organization and involving different developmental mechanisms, their sequential development follows a generic principle of recursive subdivision of space, associated with domain growth and an irreversibility condition. To investigate the morphogenesis of such hierarchical patterns, we develop a theoretical framework, based on morphoelasticity, the biomechanics of growth. In our model, the hierarchical pattern is modelled as a sum of Gaussians, each representing a subunit. The size and spacing of these Gaussians are then determined by minimizing the mechanical energy of the growing system. Our framework is simple enough to be analytically tractable, enabling us to identify the mechanisms necessary for hierarchical pattern formation and providing new insight into the developmental process involved. Our work is specifically motivated by and applied to the context of mollusc shells, in which the hierarchical ridge pattern is in some species dilated to form beautifully exuberant shell edges, which may take the form of a row of needle-like or fractal-like spines, nevertheless maintaining the hierarchical form.

## 1. Introduction

**Figure 1.**
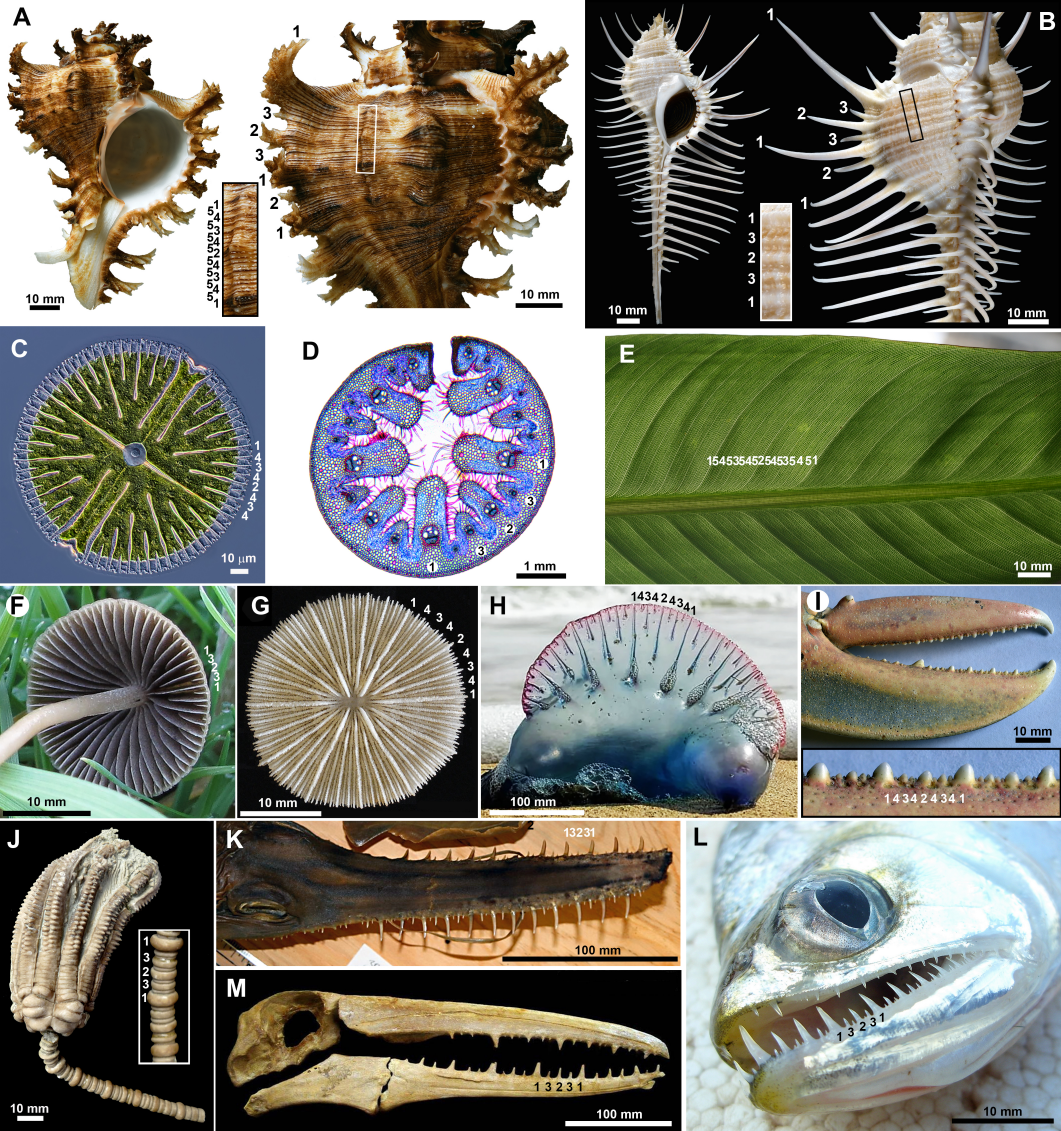
Hierarchical patterns in diverse structures in plants, fungi and animals. Spiral ridges in muricid gastropods dilated in fractal-like spines of *Chicoreus ramosus* (A) or needle-like spines of *Murex pecten* (B). In (A,B), the inset shows a zoomed-in view of the spiral ridges pattern. (C) The unicellular green alga *Micrasterias radiosa*. (D) Cross-section of a leaf of marram grass, *Ammophila arenaria*. (E) Leaf vascular network of *Strelitzia reginae*. (F) Lamellae under the cap of the Agaricomycetes *Psathyrella gracilis*. (G) Coral skeleton of the Fungiidae *Cycloseris boschmai*. (H) The siphonophore *Physalia physalis* stranded on a beach. (I) Claw of the lobster *Homarus gammarus*. (J) The fossil crinoid *Encrinus liliiformis*, Middle Triassic, Germany. (K) The sawshark *Pristiophorus cirratus*. (L) The ‘vampire fish’ *Cynodon gibbus*. (M) Skull of the fossil bird *Pelagornis* sp., Miocene, Peru. Images courtesy of (C) Rogelio Moreno (Panama); (D) Martyn L. Gorman (Scotland); (F) Sava Krstic (USA); (G) Bert W. Hoeksema (Netherlands); (H) Andreas Schwind (Germany); (J) Hans Hagdorn (Germany); (K) Museum of Comparative Zoology, Harvard University (USA); (L) Adam Carvalho (Brazil); (M) Marcelo Stucchi (Peru).

Jean-Baptiste Lamarck, the French biologist known for the first theory of evolution, wrote a treatise on invertebrate zoology in which he described and named several new species of Muricidae, a highly diversified family of marine gastropods [1]. Struck by the resemblance between some gastropods and plant leaves, Lamarck coined the following names: *Murex brevifrons* (short leaves), *M. microphyllus* (small leaves), *M. phyllopterus* (leaf-shaped wing), *M. quadrifrons* (four leaves), *M. brassica* (cabbage) and *M. endivia* (endive). He was not the only one to use such an analogy: in 1791, Johann Friedrich Gmelin, in the 13th edition of the Linné’s ‘Systema Naturae’ [2], already named the *M. cichoreus* again based on the similarity with leaves of *Cichorium endivia*, the common curly endive, and incidentally, the *M. endivia* was a junior synonym of *M. cichoreus*, i.e. the same species. Lamarck and Gmelin both saw in the fractal-like spines of this *Murex* shapes that were reminiscent of the intricately ruffled edges of endive leaves. It turned out that this resemblance is not fortuitous, the development of ruffled leaf edges and spines on seashells both resulting from a mechanical instability triggered by an excess of growth at the edge [3,4]. But while these complex shapes appear from an unpatterned initial condition in leaves, fractal-like spines of *Murex* gastropods, such as those shown in figure 1A, emerge episodically under a burst of shell secretion, dilating an already present spiral ridges pattern. The shape similarity between two completely different organisms noted by Lamarck and Gmelin does not end there. Strikingly, the spiral ridges pattern, which displays alternating lines of different heights in a robust coherent pattern, is not just found in *Murex* gastropods or even only in molluscs, but across many other organisms spanning the plant, fungi and animal kingdoms, as shown in figure 1.

The key feature of these patterns is their *size hierarchy* in which sequential levels emerge dynamically and are intercalated between previous levels. Consider, for instance, figure 1A,B. Since seashells carry the history of their morphogenesis in their shape, we see that ridges emerge sequentially during shell growth. Then, they grow in amplitude and delineate sub-domains that expand and within which new ridges emerge. The process is then repeated. In this way, the resulting hierarchical pattern records the history of its genesis and the temporal order of development.

Mathematically, this recursive space division may be described by a number series denoting both the order of appearance of each ridge and their relative amplitude. We use 1 for primary ridges of greater amplitude, which emerge first in development, 2 for secondary ridges that develop second and so on. Starting from a domain with two primary ridges that we denote 1.1, once the domain has sufficiently expanded, a secondary ridge is intercalated creating a 1.2.1 sequence, then the formation of tertiary ridges gives a 1.3.2.3.1 sequence, while quaternary ridges give a 1.4.3.4.2.4.3.4.1 sequence and so on.

A similar hierarchy is observed in other biological structures. In figure 1C–L, we display 11 other organisms where this hierarchical pattern, with intercalation of new levels following the same number sequence, is observed. In each of these systems, there is a temporal ordering, such that the 1.1 pattern will occur earlier in development than the 1.2.1 and so on, such that the pattern steps through increasing complexity as domain size increases, though the growth itself may occur continuously or not. Though the specific biological details differ significantly among these examples, and the development in some of them has been hardly studied (descriptions of the development process for each organism aside from molluscs are given in electronic supplementary material, Section S10), the widespread nature of this structural pattern suggests that the biophysical processes underlying the development of such structures might share a common dynamic rule that has been attained by many widely separated organisms.

The main objective of this paper is to investigate theoretically the morphogenesis of such hierarchical patterns. To do so, we develop a mathematical framework based on the mechanics of growing layers, with the aim of obtaining a minimal-ingredients description for hierarchical pattern formation. Our model formulation and analysis is initially focused on gastropod shells, for which much is known about the development and mechanical interactions. After demonstrating the potential for and properties of hierarchical pattern formation in this paradigm system, we examine the possibility that the same basic mechanism underlies pattern formation in the other systems.

## 2. Hierarchical ridges pattern and spines in shells

The shell of molluscs is secreted in an accretionary process by the mantle, a thin membranous elastic organ that adheres to the inner shell surface and extends slightly beyond the shell edge. At this level, the mantle takes a form incrementally recorded in the calcified shell during growth. The emergence of spines can be explained through a buckling instability of the thin elastic mantle edge that, during a growth burst, has an excess of marginal length relative to the already secreted shell edge onto which it adheres, and incrementally deforms while maintaining a shape of mechanical equilibrium [4]. This process in which a buckling instability relaxes mechanical stresses resulting from a mismatch in length is universal and explains how elastic membranes can create complex shapes in various physical and biological systems.

Unlike spines, which emerge episodically during a growth burst, the spiral ridges pattern emerges continuously during shell development, increasing in complexity as the shell edge expands. This spiral ridges pattern is present in not-closely related species belonging to all orders of gastropods, displaying spines or not, as well as not-closely related species of bivalves. The development of these ridges follows the universal ordered sequence described above, which suggests a generic behaviour of the shell-secreting system.

**Figure 2.**
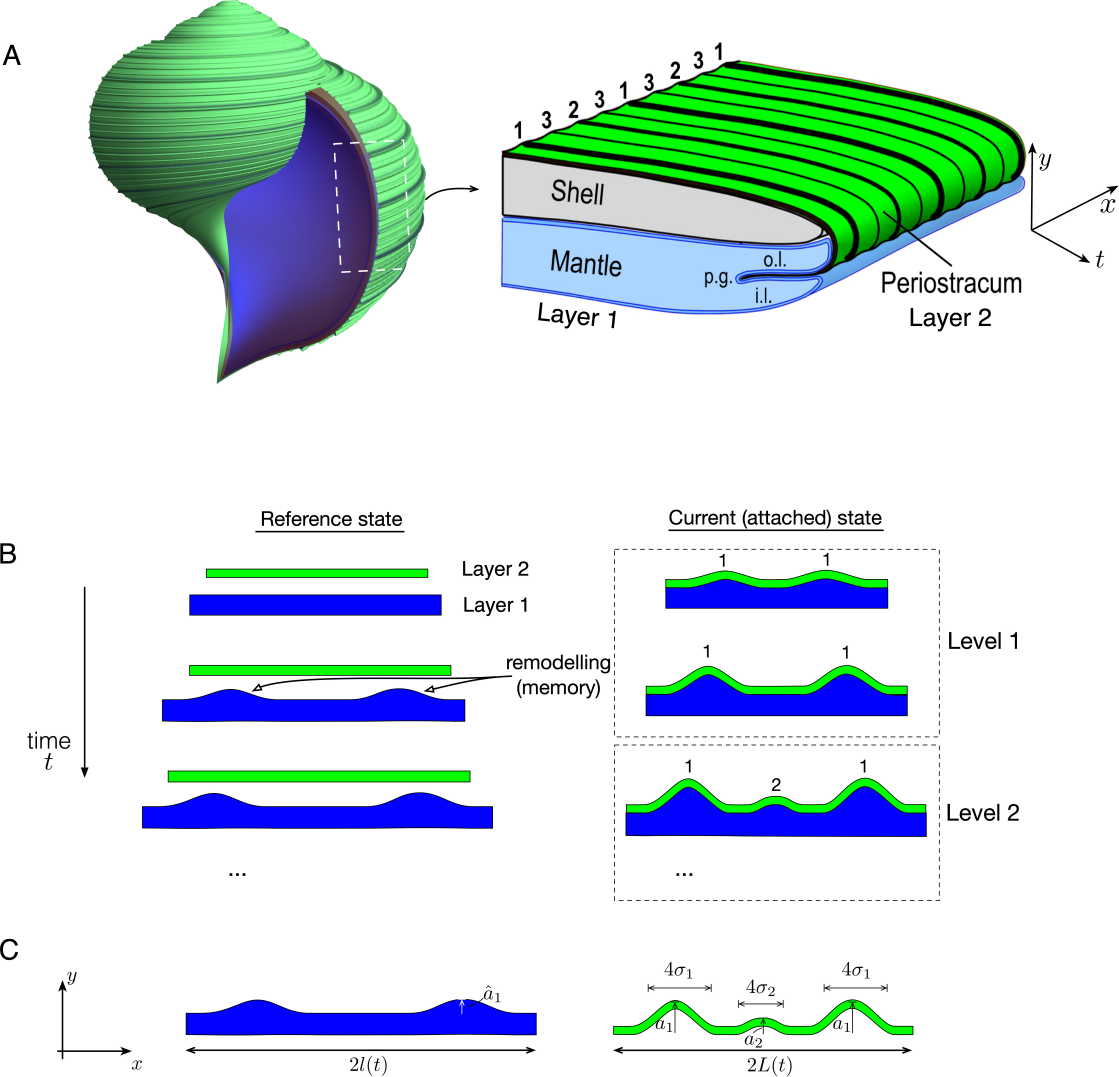
(A) Schematic of the generative zone in a gastropod. The periostracum is secreted in the periostracal groove (p.g.) and is extruded between the outer (o.l.) and inner lobe (i.l.) of the mantle edge. The hierarchical ridges pattern appears at this level and is subsequently recorded on the calcified shell during growth. In our model, the shorter periostracum is layer 2, while the longer mantle is layer 1. (B,C) Schematic of the model setup. Two elastic layers are increasing in length such that in their reference state, layer 1 has an ever-increasing excess of length with respect to layer 2, to which it is attached in the current state. The result is a bifurcation to a wrinkled state, level 1. Continued growth creates space between existing wrinkles and leads to further bifurcations to increasing hierarchical levels. Remodelling or memory is incorporated in the system through a relaxation of layer 1 towards the deformed shape. (C) Our mathematical approach consists of simplifying the mechanics of the system by restricting the shape to a series of Gaussian bumps with amplitude $a_{i}$ and width $4\sigma_{i}$.

Two ingredients can underlie the formation of this pattern in shells. First, a mismatch in length between the shell secreting mantle and the periostracum. This thin, organic layer also secreted by the mantle forms the outermost layer of the shell and isolates the supersaturated extrapallial fluid from which the calcified shell is precipitated [5]. The shape of the generative zone (the stiff periostracum on the softer mantle lobe) is incrementally recorded and fixed in the calcified shell during growth; see figure 2A. The periostracum starts to be secreted in the periostracal groove, and then moves forward while thickening and stiffening through sclerotization [6,7], a complex biochemical process of cross-linking fibrous proteins, causing close packing of the polymers, dehydration and stiffening. Lateral shrinkage of the periostracum due to cross-linking can induce continuous compressive stresses on the underlying softer growing mantle epithelium, possibly triggering a mechanical instability and folding the generative zone, whose shape is then recorded in the calcified shell.

The second ingredient is growth. During development, as the mollusc grows, so too does the secreting mantle, such that both the mantle edge and the periostracum increase in length, but always with the mantle epithelium having an excess in length with respect to the growing and shrinking periostracum. This increase in length governs the expansion of the shell, increasing the size of the domain on which the spiral ridges pattern may form. Viewing figure 2A, as shell development proceeds in the growth direction, denoted t (with increasing time), the mantle and periostracum have ever increasing lengths in the x-direction, defined following the shell edge; the length mismatch of mantle/periostracum creates a deformation in the orthogonal direction y, which is then recorded in the calcified shell. More details on shell growth and the potential role of mechanical interactions in the morphogenesis of shell patterns are provided in the electronic supplementary material, Section S1.

## 3. Modelling approach

The ridges pattern that appears on a mollusc shell is the spatio-temporal record of a wrinkling instability that evolves over the lifetime of shell growth. From a mechanical perspective, the formation of a hierarchical pattern in this system may be understood in simple terms: a length differential between two elastic layers creates a mechanical instability and wrinkling pattern; further growth of the layers expands the domain, spreading apart the initial pattern and hence generating space between the wrinkles. An increase in the length differential may amplify the original pattern, but may also generate subsequent instabilities and bifurcation to higher level patterns. The idealized system we have in mind is pictured schematically in figure 2B. We consider two elastic layers, equipped with three simple mechanisms:

(i) A length mismatch. The top layer, which we term layer 2, is shorter at all times than the bottom layer, layer 1.
(ii) A mechanical interaction between the layers. The two layers are adhered, forced to occupy the same domain, and thus layer 1 must accommodate the excess length by deforming both layers in the transverse direction. Such deformation introduces an elastic energy.
(iii) Growth. Both layers are growing, increasing in length. The growth of layer 2 represents an increase in the domain length; growth of layer 1 exceeds that of layer 2, so that the excess length increases over time, and thus the pattern develops over a growing domain. Growth is assumed to occur on a long enough time scale that the system is always in quasi-static mechanical equilibrium.

In this system, pattern formation reduces to the task of determining the time-evolution of a planar curve, characterizing the shape of the interface between the deformed layers. In the case of seashells, this idealization reflects the fact that shell growth is an accretionary process, with new shell material always occurring at the shell edge, which may be well approximated as a planar curve; the deformed shape then being recorded in the calcified shell. In this way, the final pattern appears as a surface, which may be understood to record the deformation history of a curve. Some of the other pattern forming systems displayed in figure 1 appear in a similar manner, a surface created via an advancing front, with increasing complexity appearing as the domain expands; see for instance the mushroom gills, the coral skeleton or the float of the ‘Portuguese man of war’ in figure 1F–H—the history of the pattern evolves moving radially outward. On the other hand, in systems such as teeth, pattern formation is better approximated as occurring along a single expanding curve defined by the growing jaw. In either type of scenario, we idealize the mechanism of pattern formation to the interaction of two planar elastic rods. It is also worth noting that there is a clear element of history in the system: the pattern at any given time does not form spontaneously but is strongly driven by the already existing pattern. This element of history, or memory, will form a crucial component of our model, and is shown schematically in figure 2 by a remodelling of layer 1 in response to the deformation. Our strategy is to ignore this component initially; we shall demonstrate its necessity in §4.

Even in the idealized view of planar curves, resolving the formation and time evolution of such a pattern within the framework of elasticity and continuum mechanics is a formidable task. Expressed mechanically, a typical approach would be to compute the shape of layer 2 as an evolving time sequence, treated as a planar elastic rod, whose shape at all times minimizes the sum of its bending energy and an interaction energy coming from the resistance to deformation of layer 1, and either incorporating a stretching energy or imposing a length constraint dictated by the relative growth rates. The initial wrinkling instability may be determined using standard techniques and has been investigated abundantly in engineering and biological systems [8]. However, extending beyond the initial wrinkling fundamentally requires the inclusion of nonlinearities, greatly complicating analysis, as does the resolution of growth and domain expansion. Techniques such as weakly nonlinear analysis allow one to extend into the nonlinear regime [9–11], but only slightly, and are limited by computational complexity. Fully numerical approaches such as finite element simulations have also been applied to study the post-buckling regime (e.g. [12]) and even to hierarchical dynamical buckling [13], but are computationally expensive and do not easily allow for analytical insight. Our objective is to develop a minimal mechanical model for this idealized system, in such a way that we may readily analyse the requirements for and details of a hierarchical pattern.

Letting layer 2 take the form (x,y)=(S,y(S,t)), we start from an energy of the form


(3.1) $$E[y(S,t)]:=\int_{-L(t)}^{L(t)}\frac{E_{b}}{2}{\left(\frac{\partial^{2}y(S,t)}{\partial S^{2}}\right)}^{2}+\frac{K}{2}f(y)\;\text{d}S,$$

where [−L(t),L(t)] is the expanding domain defined by growth of the shorter layer. The first term characterizes bending energy, proportional to curvature squared and with stiffness $E_{b}$ and the second term characterizes the interaction energy of the layers, with stiffness coefficient K. We consider a single term polynomial form for the interaction energy, i.e. $f=y^{m}$ for some integer m≥2. The specific value of m would depend on the material interaction. For instance, resolving the contact force on an elastic rod attached to an elastic half-space contributes a quadratic force [14], which would correspond to m=3 in our formulation. Note also that a quadratic energy, m=2, corresponds to a linear force interaction, as in the classic case of a Winkler foundation with a Hookean spring [15]. We choose to work with arbitrary m, at least initially, which will enable us to examine the role of m in pattern formation and enable for other forms of nonlinear interaction to be studied.

The energy is to be minimized subject to the length constraint


(3.2) $$\int_{-L(t)}^{L(t)}\sqrt{1+{\left(\frac{\partial y(S,t)}{\partial S}\right)}^{2}}\;\text{d}S\approx \int_{-L(t)}^{L(t)}1+\frac{1}{2}{\frac{\partial y(S,t)}{\partial S}}^{2}\;\text{d}S=2l(t),$$

where l(t)>L(t) reflects the length excess between the layers, and the linearization of curvature and arc length above is valid for small amplitudes, a valid approximation in the case of most wrinkling patterns.

Even with these minimal ingredients, determining the function y(S,t) that minimizes (3.1) subject to (3.2) constitutes a complex mechanical system. Employing the Euler–Lagrange equation yields a fourth-order differential equation for the function y. In the case m=2, the resulting differential equation is linear and may be solved exactly, though even then the problem is complicated by the nonlinear length constraint. More generally, any nonlinearity in f(y) coming from m>2 yields a system that is non-trivial to resolve even in a single time step, and analytically intractable as an evolving time sequence.

The key conceptual idea underlying our approach is to capture the essence of the process by modelling the shape of the wrinkling pattern, following the so-called ‘energy method’ of Timoshenko & Gere [16] where the general shape of an elastic solution is assumed within a family of functions with a few parameters and the energy is minimized to obtain the values of the parameters specifying the solution. We are motivated by the fact that at a local level, the structural patterns we seek to mimic have a simple ‘bump’ shape. This is certainly the case in the spiral ridges on mollusc shells, but characterizes the local shape in the other systems shown in figure 1 as well. In these systems, the complexity in structural form comes not in the shape of any individual ‘bump’, but in their hierarchical ordering and dynamic appearance on a growing domain. Our objective is to characterize the emergence of such patterns in a way that is amenable to analytical treatment. In order to exploit the simplicity in local shape, we model the pattern by a linear combination of ‘Gaussian bumps’, whose amplitude and width are to be determined. Explicitly, we assume that y(S,t) has the form


(3.3) $$y(S,t)=\sum_{i=1}^{N}a_{i}(t)\exp\left(\frac{-(S-S_{i}{)}^{2}}{\sigma_{i}^{2}}\right),\;\;S\in [-L(t),L(t)].$$

Here, the assumed form of y implies that at a local level, we model the shape of the wrinkling pattern to a particular Gaussian form defined only by the parameters $\left\{a_{i}\right\}$, $\left\{\sigma_{i}\right\}$, $\left\{S_{i}\right\}$ for i=1,2,…,N (figure 2C), rather than assuming an arbitrary function as is traditionally done in the general theory of elastic instability [17]. We note that such Gaussians are not solutions of the Euler–Lagrange equations that correspond to energy minimization, but constructing the deformed shape in this way nevertheless generates a solution that is a good approximation to the full solution, as we shall show below. Moreover, the Gaussian form facilitates an analysis of pattern formation and specifically the bifurcation to new hierarchical levels. A similar analysis could in principle be constructed with other functional forms, for instance, cosines; however, Gaussians have the desirable feature of only deforming in one direction, i.e. we get ‘for free’ the up–down symmetry breaking present in all of the patterns in figure 1.

The form (3.3) is further restricted by assuming that each level of hierarchy is characterized by the same Gaussian forms, and with the number of Gaussians determined by the numbering sequence outlined above. For instance, in the example of quaternary ridges described above, with a 1.4.3.4.2.4.3.4.1 sequence, and letting $N_{i}$ be the number of Gaussians at level i, we have $N_{1}=2$, $N_{2}=1$, $N_{3}=2$ $N_{4}=4$; this pattern would thus consist of two level 1 Gaussians of amplitude $a_{1}$ and width $\sigma_{1}$, one level 2 Gaussian of amplitude $a_{2}$ and width $\sigma_{2}$ and so on.

As well as specifying the shape, we also assume that Gaussian bumps are isolated from each other, in a way that we define explicitly in electronic supplementary material, Section S2, so that, for instance, the bifurcation to level 2 does not appear until there is sufficient space (to be justified below). The assumed form of non-overlapping Gaussians brings a huge computational advantage. As shown in electronic supplementary material, Section S2, the integrals for the mechanical energy and length constraint can be explicitly computed. For instance, the bending energy $E_{b}$ simplifies as


(3.4)Eb=Eb2∫−L(t)L(t)(∂2y(S,t)∂S2)2dS(3.5)≈Eb2∑i=1Nai2∫−∞∞[∂2∂S2(exp⁡(−S2σi2))]2dS,

where the integration domain may be extended to the full real line under the assumption that each Gaussian fits in the domain, as explained further in electronic supplementary material, Section S2. Thus, expanding the derivative, the Gaussian integrals may be computed explicitly, yielding


(3.6) $$\begin{array}{lcr} & E_{b}=\frac{E_{b}}{2}\sum_{i=1}^{N}\frac{3\sqrt{\pi }a_{i}^{2}}{\sqrt{2}\sigma_{i}^{3}}. & \end{array}$$

A similar reduction may be applied to the length constraint and interaction energy, and thus the problem reduces from minimizing a functional subject to a nonlinear integral constraint to minimizing an algebraic expression on the $\left\{a_{i}\right\}$, $\left\{\sigma_{i}\right\}$ subject to an algebraic constraint. We summarize the key steps here, while full details on the computation are provided in electronic supplementary material, Section S2. At hierarchical level 1, the energy may be expressed in the form


(3.7) $$\begin{array}{lcr} & E_{1}(a_{1},\sigma_{1})=N_{1}\left(\frac{a_{1}^{2}}{\sigma_{1}^{3}}+\mu a_{1}^{m}\sigma_{1}\right). & \end{array}$$

The first and second terms are, respectively, the reduced form of bending and interaction energy, while μ is a dimensionless constant that characterizes the relative strength of interaction energy and bending stiffness (additional constants have been absorbed as outlined in electronic supplementary material, Section S2). The length constraint can be written


(3.8) $$\begin{array}{lcr} & N_{1}\frac{a_{1}^{2}}{\sigma_{1}}=2\delta (t), & \end{array}$$

where δ(t) describes the excess length between the layers and is taken as a known input. The first key result comes from solving (3.8) for $a_{1}$, inserting into (3.7), and minimizing. This gives $\sigma_{1}$ as a function of time through δ, with the result that $\sigma_{1}$ is a slowly varying function of time, roughly constant. From this, we add an additional simplifying assumption that the $\sigma_{i}$ do not change in time—once the width of the Gaussians at a given hierarchical level are set, they remain fixed as the pattern develops, a reasonable approximation in light of the patterns in figure 1, and an assumption that further reduces the algebraic complexity.

Having fixed $\sigma_{1}$, the energy at the initial buckling is found to be a monotonically decreasing function of the mode $N_{1}$, and thus the number of Gaussians at level 1 is determined as the smallest integer for which all of the Gaussians fit in the domain. The amplitude of the level 1 Gaussians is then fully determined as a time series via (3.8). Note that if δ(t) is a linear function, meaning that the excess length increases at a constant rate, the amplitude has the square root form typical of a post-buckling analysis.

The energy at hierarchical level 2 has the form


(3.9) $$E_{2}(a_{1},a_{2};\sigma_{1},\sigma_{2})=\frac{N_{1}a_{1}^{2}}{\sigma_{1}^{3}}+\frac{N_{2}a_{2}^{2}}{\sigma_{2}^{3}}+\mu \left(N_{1}a_{1}^{m}\sigma_{1}+N_{2}a_{2}^{m}\sigma_{2}\right),$$

which is to be minimized subject to the length constraint


(3.10) $$\begin{array}{lcr} & N_{1}\frac{a_{1}^{2}}{\sigma_{1}}+N_{2}\frac{a_{2}^{2}}{\sigma_{2}}=2\delta (t). & \end{array}$$

Our assumption, as noted above, is that $\sigma_{1}$ is already determined from the initial buckling, and that $\sigma_{2}$ is also a fixed parameter, to be determined. The combination of fixed $\sigma_{i}$ and the requirement that the Gaussians fit in the domain leads to an equation defining $\sigma_{2}$ in terms of $N_{1}$, $N_{2}$, $\sigma_{1}$ and $t_{2}$, where $t_{2}$ is the time at which the bifurcation to level 2 occurs (to be determined). Effectively, it is the condition that at the point when the pattern bifurcates to level 2, $N_{1}$ Gaussians of width $\sigma_{1}$ and $N_{2}$ Gaussians of width $\sigma_{2}$ will perfectly fit within a domain whose width is defined by the bifurcation time $t_{2}$. Then, the length constraint may be solved for $a_{1}$, and inserted into the energy equation (3.9), leaving a polynomial equation for $a_{2}$ that may be minimized algebraically, and yielding an exact solution at least in the cases m=2, m=3 and m=4. Further, setting $a_{2}=0$ gives an expression that may be solved for $t_{2}$, and thus the full pattern at level 2 may be resolved analytically.

Similar ideas may be applied to determine the bifurcation time and amplitude curves for level 3 and beyond; indeed we find that explicit expressions for the amplitude curves $\left\{a_{i}(t)\right\}$, widths $\left\{\sigma_{i}\right\}$ and the bifurcation times $t_{i}$ at which the next hierarchical level appears, may be computed up to at least level 5 in the case m=4, though naturally the algebraic expressions quickly grow in complexity. Note that the ordered number sequence noted in the introduction, e.g. 1.1 to 1.2.1 to 1.3.2.3.1 when starting from $N_{1}=2$, emerges as a natural consequence of uniform growth, which uniformly creates space between each existing Gaussian so that at the next bifurcation, new Gaussians will fit in between each pair of existing Gaussians.

### 3.1. Validation

**Figure 3.**
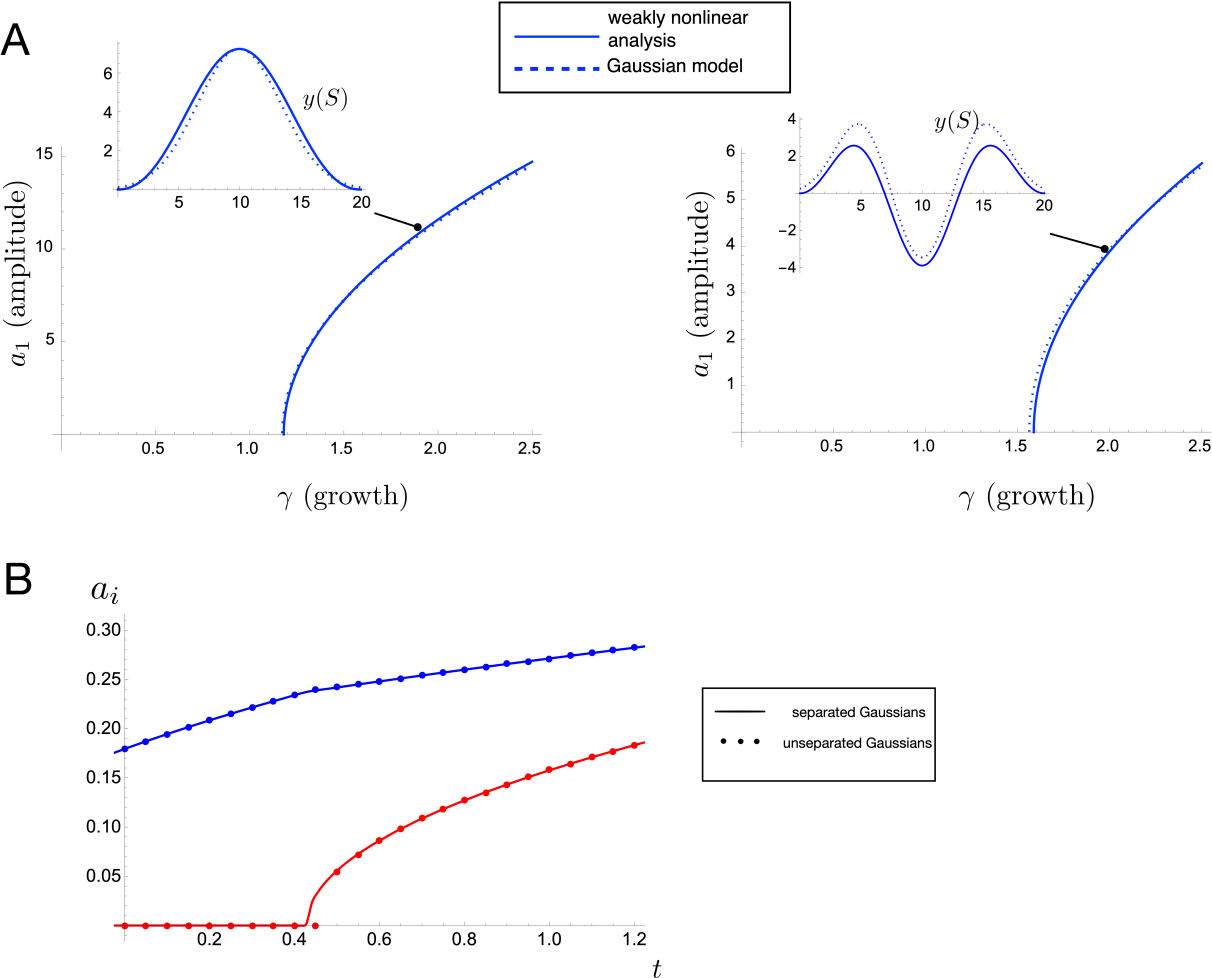
Validation of the Gaussian bumps approach. (A) Comparison of initial amplitude curves for Gaussian model and weakly nonlinear analysis of growing elastic rod [9], both for $N_{1}=1$ (left) and $N_{1}=3$ (right). The parameter γ characterizes the length excess as defined in electronic supplementary material, Section S3. Inset plots show the buckled shape at the indicated points. (B) Amplitude curves for levels 1 and 2 as computed via separated Gaussians (solid curves) and numerically computed energy minimum without assuming separated Gaussians (dots). The level 1 curve has amplitude $a_{1}$ in blue, and level 2 has amplitude $a_{2}$ in red. Parameter values provided in electronic supplementary material, Section S9.

In order to validate the ‘Gaussian bumps’ approach, we conduct two separate comparisons. First, we compare amplitude curves and resulting shape of the initial wrinkling instability between our approach and the weakly nonlinear analysis presented in [9]. In the latter, a post-buckling analysis of a growing planar elastic rod linearly attached to a foundation is conducted. This is equivalent to our system with m=2, and with an increasing excess of length but no growth of the domain. The relative strength of the foundation is captured by a single parameter, which determines the buckling mode. Specific details on this comparison are provided in electronic supplementary material, Section S3. The result appears in figure 3A, plotted with two different choices of foundation parameter that correspond to a mode 1 (left plot) and mode 3 (right plot) buckling instability. In each case, our reduced model gives almost identical amplitude curves to the more complex computations, with no fitting parameters.

As a separate form of validation, we relax the simplifying assumption that Gaussians do not overlap. We maintain the assumption that the deformed shape consists of a sum of Gaussians, but allow them to overlap. We then compute the amplitude curves and bifurcation point for the first two hierarchical levels. Details are again provided in electronic supplementary material, Section S3. The resulting comparison appears in figure 3B, again showing excellent agreement.

These comparisons provide strong support for the Gaussian approach, which thus provides a flexible and tractable tool to study pattern formation well beyond the first bifurcation point. Here, it is worth stressing the computational advantage: in the weakly nonlinear analysis, the amplitude is limited to level 1, and requires a detailed computation involving a second-order asymptotic analysis of a six-dimensional nonlinear differential equation boundary value problem. Our approach enables to produce almost identical amplitude curves with a few lines of algebra, that are also easily extended past the validity of the weakly nonlinear regime.

## 4. Results

### 4.1. The role of memory and nonlinearity

**Figure 4.**
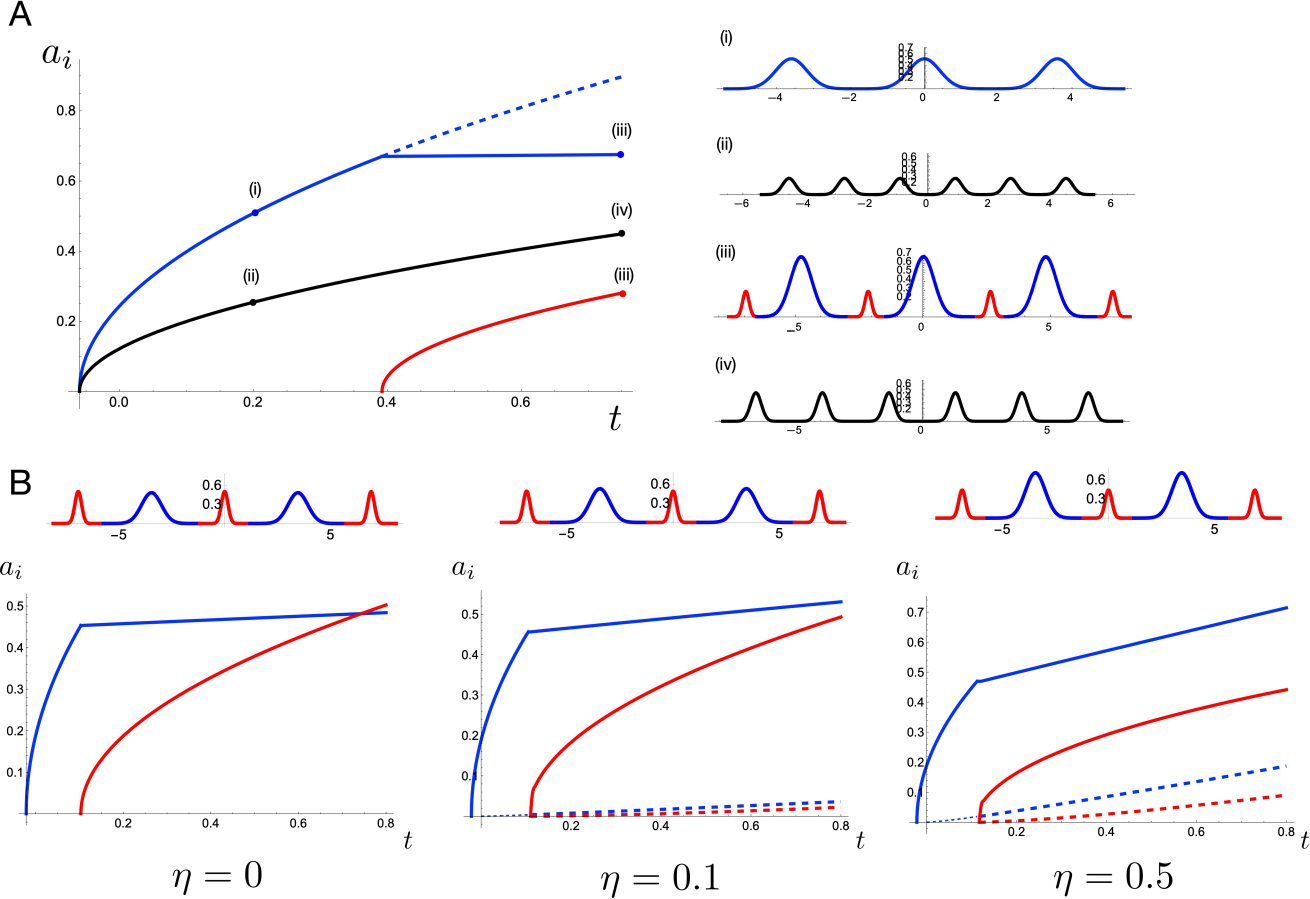
(A) Amplitude curves for a quadratic interaction energy (black) versus a quartic interaction energy (blue and red). For the quartic energy, the blue curve represents the level 1 amplitude and the red curve level 2. Profiles at the indicated points are shown at right. (B) The impact of memory on the evolution. Level 1 (blue) and level 2 (red) amplitude curves are shown for varying relaxation rate η, with profiles at t=0.8 shown above. The dashed blue and dashed red curves are the amplitude curves for ${\hat{a}}_{1}$ and ${\hat{a}}_{2}$, respectively. Parameter values provided in electronic supplementary material, Section S9.

Before comparing the hierarchical patterns produced from our theoretical approach with observations on mollusc shells or other organisms, we first analyse key features of pattern formation. In figure 4A, we show amplitude curves plotted as functions of time for two different forms of interaction energy: $f(y)=y^{2}$ (black) and $f(y)=y^{4}$ (blue and red).

Focusing first on the latter case, the time at which the red curve (level 2 amplitude) crosses the axis represents the bifurcation time $t_{2}$, the appearance of the level 2 pattern. Beyond this point, some of the excess length goes to the level 2 Gaussians, and thus the level 1 amplitude (blue curve) increases at a lower rate than if level 2 did not appear (shown by the dashed blue line). The situation is significantly different in the case of $f(y)=y^{2}$. First, the change in form of interaction energy impacts the mode of the initial buckling, so that $N_{1}=6$ Gaussians appear in the initial wrinkling compared to only $N_{1}=3$ with $f(y)=y^{4}$. More importantly, this case does not undergo a bifurcation to level 2, a fact that we algebraically show generally holds for a quadratic interaction energy (see electronic supplementary material, Section S2). A quadratic energy corresponds to a linear resistive force applied by layer 2 to layer 1, and we conclude that for this system, *a linear force does not produce a hierarchical pattern*, suggesting that nonlinearity in the interaction force is a necessary ingredient to generate a hierarchical structure. The necessity of nonlinearity for generating more complex wrinkling patterns is consistent with previous work [14] and indeed the relevance of nonlinear effects in the instability of elastic beams was noted as early as 1937 in the classic work of Biot [15].

While the model so far is able to explain the appearance of new hierarchical levels by assuming a nonlinear interaction force, it is not sufficient to explain the full evolution of a system in which stress relaxation takes place. In particular, the system so far is memory-less, which can lead to unphysical patterns. For instance, the left plot of figure 4B shows level 1 and level 2 amplitude curves with a choice of parameters such that the amplitude curve for level 2 (red curve) crosses above that of level 1 (blue curve). In this ‘memory-less’ system, it is energetically favourable for the secondary pattern to grow larger than the primary pattern, a feature that is not observed in any hierarchical patterns in nature, to our knowledge. This effect of ‘hierarchy breaking’ may be resolved by incorporating a memory into the system modelled by a local remodelling of the layers in response to deformation, so that the interaction energy is relieved via a slow relaxation towards the deformed shape. To include such a remodelling mechanism, we change the form of the interaction energy from $f(y)=y^{m}$ to $f(y)={\left(y(S,t)-\hat{y}(S,t)\right)}^{m}$*,* where $\hat{y}(S,t)$ describes locally the shape of layer 2, and gives a potentially non-flat shape at which the interaction energy is minimized. A natural choice is to evolve the shape of $\hat{y}$ towards the shape of y, for which we impose an evolution law


(4.1) $$\begin{array}{lcr} & \frac{\partial \hat{y}(S,t)}{\partial t}=\eta \left(y(S,t)-\hat{y}(S,t)\right) & \end{array}$$

where 1/η is the typical time scale at which stress is relaxed through remodelling.

Within the Gaussian framework, the function $\hat{y}$ has the same profile as y, i.e. a sum of separated Gaussians, and with the same widths $\sigma_{i}$ as y, differing only in the amplitudes, denoted ${\hat{a}}_{i}$, and the remodelling law (4.1) thus translates to an evolution law for ${\hat{a}}_{i}$ (see electronic supplementary material, Section S4).

The middle and right plots in figure 4B show the amplitude curves for η=0.1 and η=0.5, respectively. Here, the dashed lines plot the amplitude curves for ${\hat{a}}_{1}$ and ${\hat{a}}_{2}$ (blue and red dashed, respectively). Increasing η means that the ${\hat{a}}_{i}$ relax to the $a_{i}$ more quickly. This has the effect of diminishing the energetic cost of further increases in amplitude, which ultimately serves to preserve the ordering of amplitude by hierarchical level. Thus, we add memory as the third ingredient for order-preserving hierarchical patterns. This mechanism is known to be present in the mantle/periostracum system, in which the mantle epithelium is compressed and irreversibly remodelled due to deformation at the location of the ridges, thus changing the form of the mechanical interaction (see electronic supplementary material, Section S1).

### 4.2. Pattern variation

The analysis above shows that both the interaction energy exponent m and system memory rate η are important parameters for the existence and structure of a hierarchical pattern. Here, we demonstrate the impact of two other key parameters on the resulting pattern. One is the relative growth rates of the layers, which dictates the excess length of layer 1 and the rate of domain expansion. By non-dimensionalizing time based on the growth rate of layer 2, the excess length increases at a rate characterized by the dimensionless parameter g. Second, the relative strength of interaction stiffness (characterized by the parameter K) to bending stiffness $E_{b}$ determines the relative cost of deviation from $\hat{y}$ to the cost of curvature. This effect is characterized by a single dimensionless parameter, denoted μ, whose specific definition, along with that of g, is given in electronic supplementary material, Section S2.

**Figure 5.**
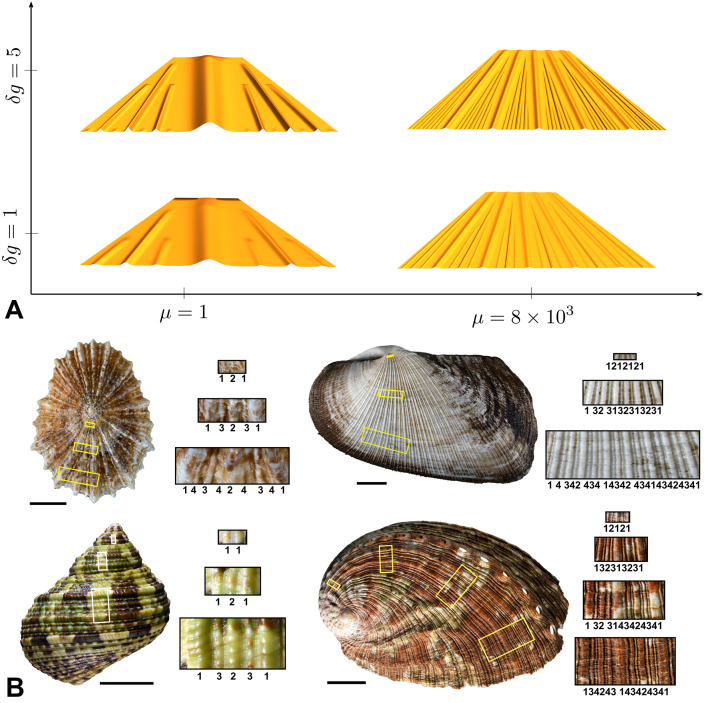
(A) A morphospace of hierarchichal pattern with varying differential growth rate g and stiffness ratio μ. A larger value of μ generates a denser pattern, while an increased growth rate leads to an earlier bifurcation to new hierarchical levels. Parameter values provided in electronic supplementary material, Section S9. (B) Variation of hierarchical ridge patterns in mollusc shells (gastropods and bivalve). From top left to bottom right *Patella tabularis*, *Trisidos semitorta*, *Turbo intercostalis*, *Haliotis tuberculata*. Scale bars, 10 mm.

In figure 5A, we show a morphospace of the patterns for four different values of the pair {μ,g} (all other parameters taking the same fixed values, provided in electronic supplementary material, Section S9). A larger value of g corresponds to a higher rate of increase of excess length between the two layers, such that the bifurcation to new hierarchical levels occurs at an earlier time. A larger value of μ corresponds to a greater relative energy cost for deviation of y from $\hat{y}$ compared to bending energy (conversely, e.g. μ=0 if there is only bending energy). This stiffness ratio dictates the density of pattern such that $N_{1}$, the number of Gaussians appearing at level 1, increases with μ.

This analysis shows that both the density and hierarchical level attained may be fine-tuned in a given biological system by varying the relative growth rates and/or material properties. In figure 5B, we show shells from four different species of mollusc, exhibiting a qualitatively similar variation in density and hierarchical levels.

## 5. Needle-like versus fractal-like spines

Next, we consider the intricate and fascinating spine patterns observed in certain gastropod species. These spines appear as the dilation of an already present spiral ridges pattern following a burst of commarginal mantle growth. The spiral ridges pattern has the hierarchical form already described by our mathematical model. However, the dilation from ridges to spines can introduce significant complexity to the pattern. In particular, there is a marked difference between the form of spines of shells such as *Chicoreus ramosus* and those of shells such as *Murex pecten* as shown in figure 1A,B. The spines on *Murex pecten* are separate from each other, reaching significant height and a needle-like appearance. We refer to this pattern as linear spines, as the spines are mutually separated and nearly aligned along the shell margin. Conversely, the spines of *Chicoreus ramosus* have a fractal-like structure, with smaller spines appearing on the side of larger spines, and occasionally another level of even smaller spines emerging from their side.

Despite these clearly distinct patterns, both shell types share the same fundamental characteristics. Another notable feature is that the hierarchical structure is maintained in the dilation. For instance, in *Murex pecten*, the tallest spines are dilated from the level 1 ridges, and between these are level 2 spines dilated from level 2 ridges, with level 3 spaced in between each 1 and 2 and so on. For fractal-like spines, the hierarchical ordering is also maintained, but with a more complex form. The peak of a level 1 (tallest) spine can be traced back to a level 1 ridge, and the peak of a level 2 spine can be traced back to a level 2 ridge. The difference is that the higher level ridges do not appear as separate spines, but as fractal-like protrusions emerging from a lower level spine. On *Chicoreus ramosus*, for instance, it is typical to find a level 4 ridge dilated on the side of a level 1 spine. The fractal order may go further, with a level 6 ridge dilating on the side of a level 4 spine, itself dilated on the side of a level 1 spine. Clearly, within a fractal-like structure, many different patterns are possible; however, the hierarchy of the ridges is maintained both in the sequence of dilation (level 1 ridges dilate before level 2), and in the spine amplitude. Further, for linear spines, the spine width is similar to the ridges width, while this does not hold generally for the fractal-like pattern. Indeed, by construction, the width of the level 1 spine must be significantly wider than the level 1 ridge; this is a geometric requirement as the level 1 spine contains a number of higher level ridges that dilate from its side.

This last fact is key to understanding the two forms. The ridge pattern on a shell that undergoes fractal dilation has a much higher density, and with decreased ridges width compared to shells that undergo a linear dilation. We have shown above that significant differences in width and density may be brought about by changes in material parameters. Our hypothesis is therefore: *Given a burst of growth of the mantle, a less dense ridges pattern is mechanically driven to a linear pattern of spines, while a more dense ridges pattern mechanically generates a fractal-like spine pattern*.

To test this hypothesis, we adapt our modelling framework to study *ridge dilation*. We first note a difference in the biophysical process: ridges form due to sclerotization and shrinking of the thin periostracum in contact with the thick and soft mantle. Within our modelling framework, the periostracum corresponds to the shorter layer 2 while the mantle, or at least the outer lobe of the mantle, is the longer layer 1. Following [4], the spines pattern is due to a burst of growth (or at least length increase) of the whole mantle and periostracum, and the pattern forms due to the interaction with the calcifying shell edge. Therefore, we apply the same modelling framework, but using now the mantle/periostracum as layer 1 (implying a significant change in material properties), with an increasing length excess in comparison to the calcifying shell edge, which provides resistance to deformation and thus plays the role of layer 2. The burst of growth corresponds to a very large value of g, but the domain width is nearly constant; that is, in (3.2), l(t) increases rapidly while L is fixed.

### 5.1. Fractal-like Gaussians

**Figure 6.**
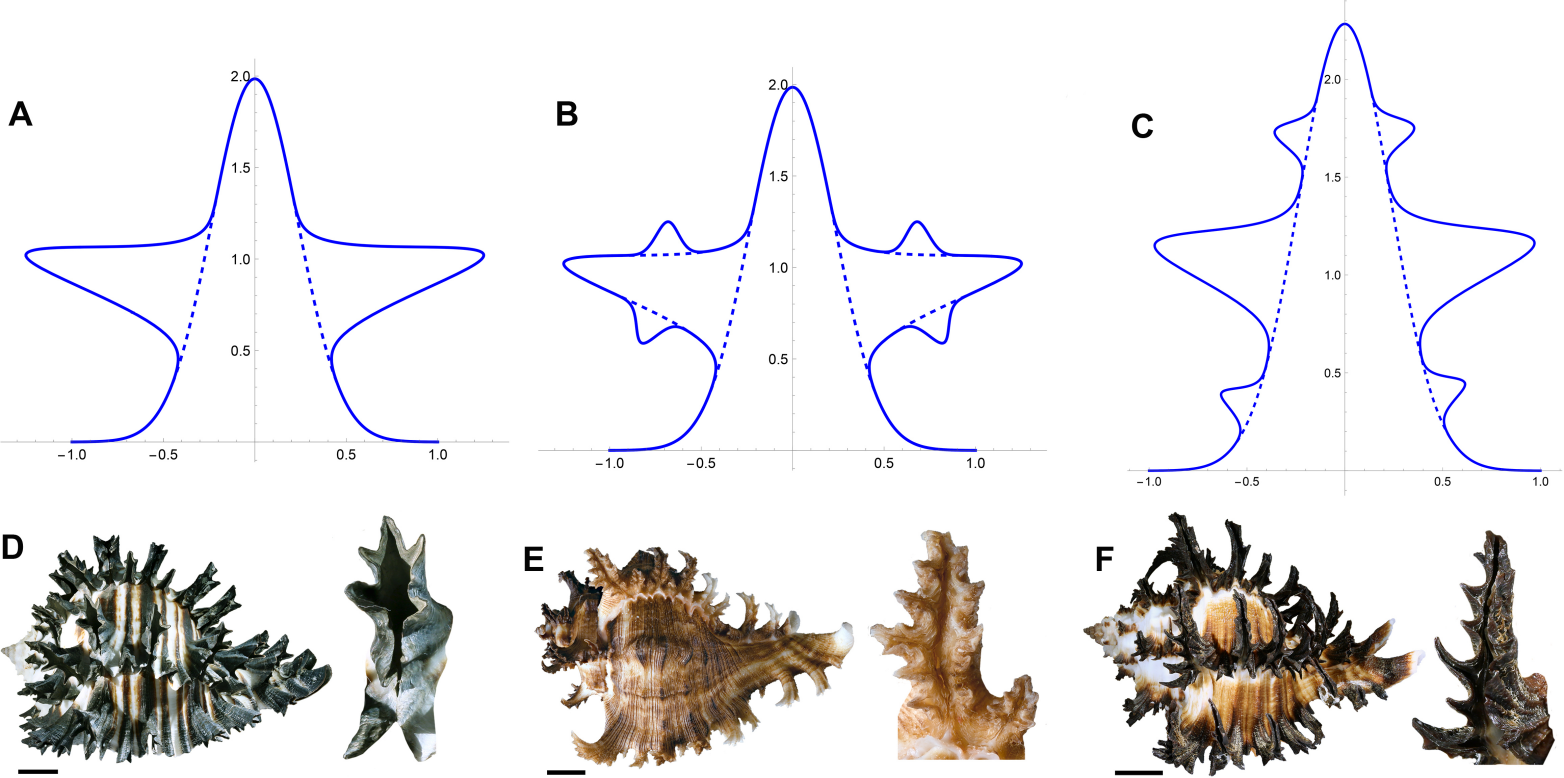
(A–C) Fractal formation within Gaussian approach. (A) Gaussians appear on the side of an existing Gaussian. (B,C) Two alternative developments of the pattern in (A) to more complexity. (D–F) Fractal-like spines in Muricidae: (D) *Muricanthus radix*; (E) *Chicoreus ramosus*; (F) *Hexaplex cichoreum*. Scale bars, 10 mm.

We must also adapt our model to describe fractal-like structures. Remarkably, our Gaussian-bump construction extends to the more complex patterns exhibited in a fractal-like structure. The key idea underlying this extension is that such structures may be approximated by locating new Gaussian bumps on the sides of existing Gaussians. This idea is shown schematically in figure 6. In figure 6A, a Gaussian appears on the side of an existing Gaussian with amplitude $a_{1}$ and width $\sigma_{1}$. In figure 6B, the fractal order is extended. In figure 6C, an alternative bifurcation to higher level is shown. This highlights how extending from a pattern of linear Gaussians to a fractal structure creates an increase in the number of potential forms, to the extent that even classifying levels requires care. And indeed, such diversity can be seen in gastropods exhibiting fractal-like spines—some examples appear in figure 6D–F.

Despite the complex geometry of a fractal-like structure, and the fact that the shape can no longer be described by a graph y(S,t)*,* by constructing fractal-like structures via new Gaussians appearing on the edge of existing ones, the total mechanical energy can still be constructed by summing the energy contributions of each individual Gaussian. In this way, the evolution of the system reduces to algebraic computations similar to the linear Gaussians case. The main computational difference with a fractal-like structure relates to the condition of when sufficient space exists for a new Gaussian to appear—since the ‘domain’ for a new Gaussian is the side of an existing one, the bifurcation condition for sufficient space is framed in terms of the amplitude of the existing Gaussian. We provide full details on the Gaussian energy approach for fractal-like structures in electronic supplementary material, Section S5, including detail on the geometry of plotting the resulting pattern.

### 5.2. Ridges density as a predictor for linear versus fractal-like spines

**Figure 7.**
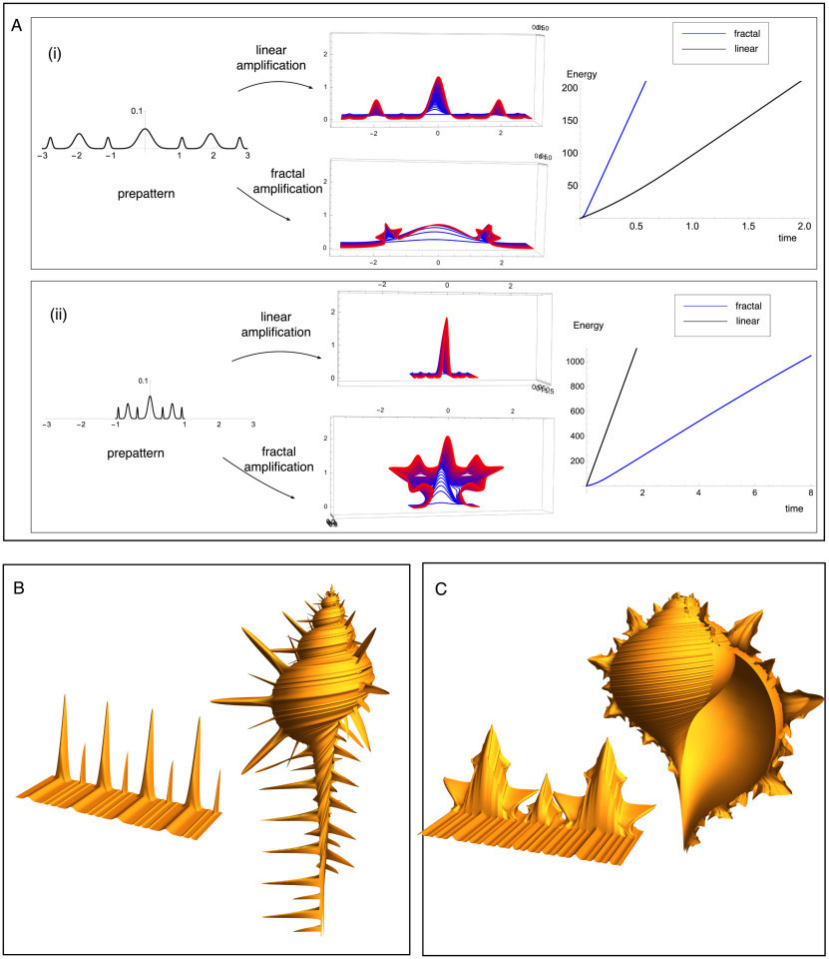
Fractal-like versus linear dilation of ridges to spines. (A) Energy minimizing dilations of a pre-existing hierarchical ridges pattern with low (i) and high (ii) density of ridges are computed both for linear and fractal-like patterns, with the resulting spines pattern appearing in the middle column. The total mechanical energy in each case is plotted against developmental time at right, showing that in the low density case, the linear pattern is mechanically favourable, while the fractal-like pattern has lower energy in the high density case. (B,C) Realistic surfaces or ridges dilated to spines are generated as mechanically predicted outputs, with parameters for which the linear spines occur in (B) and the fractal-like spines occur in (C). The ridges and spine patterns are then combined with a parametrization of a shell to produce the shell simulations at right. Parameter values provided in electronic supplementary material, Section S9.

To investigate whether ridges density can serve as a predictor for dilation to linear versus fractal-like spines, we first construct two different ridge patterns—one with a lower density of ridges, and one with a higher density of ridges—by placing the same pattern 3.2.3.1.3.2.3 on a domain width 2L with L=3 and L=1 (see left panels of figure 7A). Keeping L fixed, we increase l(t) and generate both linear and fractal-like spines as energy minimizers. For the linear spines, the width at each level is taken equal to the width of the corresponding ridge. For the fractal-like spine, the width of the level 1 spine is instead equal to the *domain width*, with the level 2 spine bifurcating from the side of the level 1 spine, and the level 3 spine bifurcating from the side of level 2, when sufficient amplitude is reached, leading to the middle panels of figure 7A.

In each case, we compute and compare the total energy as a function of time, with the energy appearing in the right panels of figure 7A (for more details and parameter values, see electronic supplementary material, Section S6). In the low density case, figure 7A(i), the linear spines are energetically favourable, while the fractal-like spines have a shape distinctly different from any mollusc shell, and a higher energy due to the much wider level 1 spine. In the high density case, figure 7A(ii), on the other hand, the fractal-like spines have a more realistic shape and indeed have lower energy than the linear spine. Here, the bifurcation to level 2 amplification never occurs. To understand this result intuitively, we note (see electronic supplementary material, Section S2) that for a single Gaussian with amplitude a and width σ, the arc length scales with $a^{2}/\sigma$, the bending energy is proportional to $a^{2}/\sigma^{3}$ and the substrate (interaction) energy is proportional to $\sigma a^{4}$. When the density of ridges is high, σ is small, and the bending energy is thus much higher for the linear spines compared to the fractal spines. For a lower density and thus larger σ, the bending energy for the linear spines is smaller, while the length constraint leads to a higher amplitude and thus higher substrate energy for the fractal-like spines. Comparing again the shells in figure 1, our analysis offers a clear explanation: the ridges of *Chicoreus ramosus* are so close together that a dilation to needle-like spines would come at too high a cost of bending of mantle/periostracum to be mechanically possible.

### 5.3. Mechanically patterned shells

Having demonstrated the difference that ridges density has on the amplification to spines, we also show that realistic shells of both types can be constructed as output of our model. The simulated shells appearing in figure 7B,C are each mechanically predicted: both the ridges and spines patterns are solutions of our model, i.e. energy minimizers within our two-layer Gaussian reduction. In both cases, we have produced first the ridges pattern, and then amplified the ridges pattern to the energy minimizing spine pattern, leading to linear spines on the left and fractal-like spines on the right. The key difference underlying the two is the density of the ridges pattern, which we have varied by using different values of the parameter μ. For the shell on the right, we have used a larger relative value of μ, leading to a denser pre-pattern that is dilated to a fractal-like spine pattern, while the shell on the left has a lower value of μ, and thus a less-dense ridges pattern that is dilated to a linear spine pattern upon a burst of growth. The ridges and spines patterns are initially produced as surfaces (shown at left), which are then mapped onto a full three-dimensional shell simulation to produce the shell images on the right. The shell geometry is not directly feeding into the mechanical calculation; rather, we are dressing a smooth shell with the mechanically computed ridges/spines pattern. It is worth noting, however, a difference in geometry between the two shells (i.e. before the spines/ridges are included): the shell in figure 7B has a lower aperture expansion rate than figure 7C. A higher aperture expansion rate would correspond to an increased g, thus creating an increased ridges density that favours the fractal-like dilation, according to our framework. Simulation of the fractal-like shell in particular requires a significant architecture combining geometry and mechanics, for which complete details are available in electronic supplementary material, Section S7. These examples highlight how distinct patterns can be predicted as manifestations of the same basic physics of development with only different underlying parameters generating different energy-minimizing pathways. And indeed, recent molecular phylogenies of Muricidae show that despite major differences in shell morphology, *M. pecten* is very closely related to *Chicoreus* species [18].

## 6. A generic dynamic

The structure of the generative zone in gastropods, constituted of a stiff layer (periostracum) bound to a softer elastic substrate (the mantle) is also found in bivalves, which explains, through evolutionary convergence, the emergence of a similar hierarchical pattern. Further, a similar pattern of spiral ridges may be seen also in brachiopods (e.g. Strophomenidae), another phylum whose shell grows in an accretionary way, incrementally secreted at the margin by a thin membranous elastic mantle that secretes first a periostracum, serving as a matrix for the deposition of the calcium carbonate of the shell [19]. More surprisingly, as noted in the introduction, similar hierarchy is also observed in remarkably diverse structures in plants, fungi and animals (figure 1C–L): the cell wall of unicellular green algae, leaf venation, the lamellae of mushrooms, the gas-filled float of siphonophores, the coral skeleton, the denticles of a lobster’s claw, the stalk of crinoids, the denticles of sawsharks, the teeth of certain fishes and the pseudoteeth of a fossil bird. Investigating in detail the development of all of these structures to identify equivalent mechanisms is beyond the scope of the present work. But available studies suggest that mechanical patterning, a hypothesis still rarely considered in developmental biology, might play an important role in their emergence.

For instance, hierarchical patterns are seen in the leaf venation of some plants with parallel venation (veins running parallel to one another) such as *Ammophila* (figure 1D) or *Strelitzia*, with up to six levels of veins (figure 1E). It is also seen in other monocotyledons such as in *Musa* (banana) leaves. Despite their large variety, a characteristic of the leaf vascular network is the hierarchy of the veins' width, that records their sequential development during leaf growth. Based on the similarity to the hierarchical pattern of the networks of cracks in drying muds and gels, it has been suggested that the leaf vascular network is patterned by mechanical stresses [20]. In drying muds and gels, cracks of different width relax tension generated by shrinkage mismatches and delineate individual domains subdivided into smaller ones over multiple generations [21]. In the case of leaves, a growth mismatch between the outer epidermis, and the inner faster-growing compressed mesophyll in which veins appear, is the driving force regulating the differentiation of vascular network. Numerical models based on this hypothesis reproduce many features of the network geometry [22,23]. Current studies favour the mechanical hypothesis, and suggest that the formation of vascular network can be described in terms of auxin-dependent specification of future vascular cells and mechanical constraints generated at the tissue level [24].

**Figure 8.**
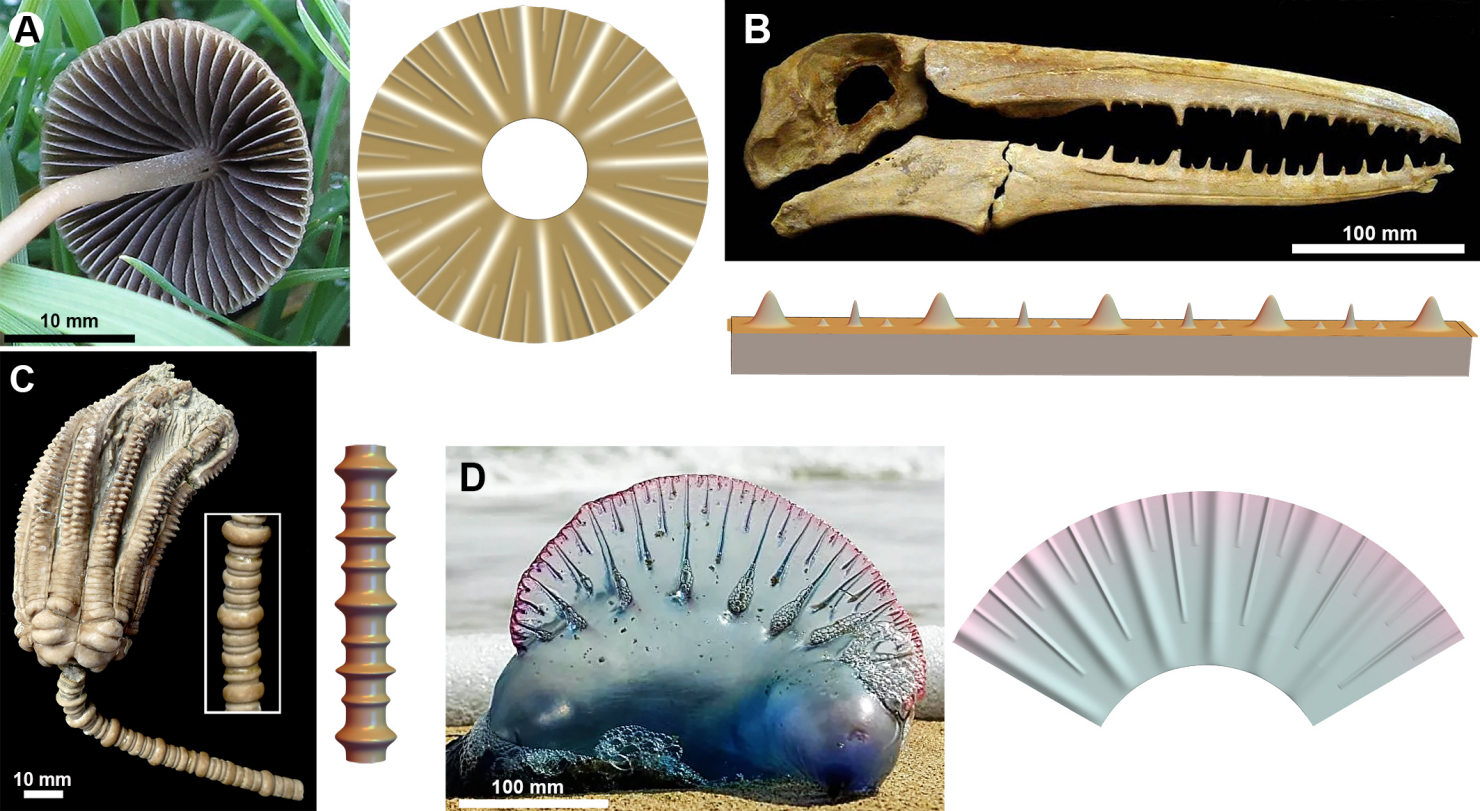
Simulation of the hierarchical pattern seen in (A) the lamellae under the cap of a mushroom, (B) the pseudoteeth of the fossil bird *Pelagornis*, (C) the columnals of the stalk of a fossil crinoid and (D) the septa of the pneumatophore of the ‘Portuguese man of war’. Parameter values provided in electronic supplementary material, Section S9.

Concerning the other organisms displayed in figure 1, a mechanical explanation similarly provides a reasonable hypothesis underpinning the hierarchical nature. In electronic supplementary material, Section S10, we outline the possible interpretations for each of them within our growing two-layer idealization. We also demonstrate that if we adopt such an interpretation, then simulating this diversity of patterns is straightforward. In figure 8, we present examples in which our model is used to simulate the hierarchical patterns from four of the organisms in figure 1. This highlights the adaptability of the modelling approach. Converting the pattern to a different geometry such as an expanding circle, for instance, requires only a minor modification (more details provided in electronic supplementary material, Section S8). While the model may be able to reproduce these patterns, this does not constitute proof of a dynamic mechanism. That is, we cannot claim to have resolved the developmental mechanism of 11 different widely separated organisms, though our analysis provides a conceptual pathway in which a more detailed investigation of any of those organisms could be explored.

In the context of mollusc shells, we have shown how examination of the mechanical and geometric parameters for which a fractal versus linear spine may be predicted provides a biophysical basis through which we may understand the difference, and similarity, between morphologically distinct species such as *M. pecten* and *C. ramosus*. The ability of our framework to reproduce particular details of hierarchical patterns in other organisms suggests, through appropriate interpretation of model parameters in terms of the specific developmental biology of a given organism, a powerful platform from which we may gain insight into the origin and evolution of such patterns. Still, we must acknowledge that our model was constructed under several simplifications, and there is certainly room for model improvements in future work. The use of non-overlapping Gaussians in a modular energy formulation brought significant computational and analytical advantage, and is reasonably justified in light of our validation comparisons and structural features observed in real systems. However, these modelling choices would benefit from a more fundamental derivation, e.g. reduction from a full three-dimensional elastic system, and/or comparison with detailed computational simulations such as finite elements. More explicit biology on the growth process could also be incorporated, though there is a trade-off between biological complexity and model tractability. The combination of a simplified approach as we have presented here and a more complex system would provide a powerful tool for both qualitative and quantitative analysis of hierarchical pattern formation.

## 7. Self-similar origami

Morphogenesis does not only depend on patterns of gene expression and molecular signalling pathways but also involves mechanical forces and tissue deformations that physically shape organisms in three-dimensional geometries. Origami, the Japanese art of paper folding, is sometimes used as a metaphor [25,26] to emphasize that fairly simple processes of sequential tissues folding during development underlie the emergence of complex and extremely diverse shapes of living beings. On the other hand, it is well known that a recursion of simple equations can produce unexpectedly complex fractal patterns [27]. Developmental processes are clearly more complex than origami, and true fractals are limited to mathematics. Yet, it is remarkable that such a complex fractal-like spiny edge in gastropods can be generated by a simple process of sequential folding of the generative zone during development, an episodic buckling instability of the generative zone dilating a spiral ridges pattern emerging recursively during development. While in origami the reproducibility of shapes depends on a precise sequence of predefined steps, the spiral ridges pattern does not rely on a pre-programmed succession of folds, but is built on itself through a self-organized folding sequence. Its development goes through sequential bifurcations, folds generated within a propagating front (the shell edge) by the generative zone creating the conditions for the next bifurcation and selecting ever higher-order modes in a robust way.

The same hierarchy characterizes strikingly different structures in organisms belonging to three different kingdoms, at widely different scales (micrometre to centimetre, i.e. spanning a factor of 10 000), different levels of biological organization (from the level of cell wall of unicellular algae to the level of organs and even whole organism), and involving different developmental processes. Though these similarities may appear coincidental, the setting up of most (maybe all) of these structures turns out to share a common dynamic. They emerge sequentially over an expanding domain, each growing sub-unit dividing a domain in expanding sub-domains within which new sub-units emerge sequentially and so on. Therefore, the spatial hierarchy of the pattern records the history of its formation and the temporal order of development of the sub-units over an expanding domain. This principle of recursive space division underlies purely mathematical fractals such as the Sierpiński triangle, a self-similar set that can be obtained by subdividing recursively an equilateral triangle into smaller ones. Among physical systems, crack patterns result from the shrinkage of a layer (e.g. desiccated mud) adhering to a non-shrinking substrate, the mismatch generating mechanical tensions that are relaxed by the formation of successive fractures subdividing recursively a domain into smaller ones. The existing cracks delimit the domain defining the boundary conditions for the mechanical stress field that governs the genesis of the future cracks, the geometry of the final pattern recording the history of its formation [21]. Interestingly, these space-dividing cracks may generate a hierarchical pattern reminiscent of the one we describe in this study [28]. One key conclusion of our paper is that mechanical instabilities can recursively divide an expanding domain and can be involved in the morphogenesis of strikingly different hierarchical structures in plant, fungi and animals, which might provide a framework for understanding their development despite the variety of mechanisms involved in each specific case.

## Data Availability

Mathematica notebooks reproducing model output are available in the public depository https://ora.ox.ac.uk/objects/uuid:39b09dba-796b-4a10-835d-e3a4dedbff82. Electronic supplementary material is available online [29].
